# Is Cervical Inlet Patch Important Clinical Problem?

**Published:** 2014-06

**Authors:** Gurol SAHIN, Gokhan ADAS, Bora KOC, Adem AKCAKAYA, Yasar DOGAN, Suha Goksel, Ozben Yalcin

**Affiliations:** 1 Ethica Hospital, Department of Surgery/Istanbul, Turkey;; 2 Bakırkoy Sadi Konuk Training and Research Hospital, Department of Surgery/Istanbul, Turkey;; 3 Bezmialem Vakif University, Department of Surgery/Istanbul, Turkey;; 4 University of Acıbadem, Medical School, Department of Pathology/Istanbul, Turkey

**Keywords:** Cervical inlet patch, endoscopy, heterotopic gastric mucosa

## Abstract

**AIM:**

In this study we aim to determine the frequency of Inlet Patch (IP) and its association to clinical symptoms and draw attention to be aware of this heterotopic gastric mucosa.

**METHODS:**

This study was a prospective case series that IP was detected in the upper gastrointestinal endoscopy. Patients with laringopharyngeal reflux symptoms underwent endoscopy between March 2009 and July 2012 in two different institutions. All the biopsies were obtained from if there is the IP lesion and antral or/and gastric mucosa. The data was prospectively evaluated. The prevalence was compared with those of patients that did not determine IP in the study period.

**RESULTS:**

3907 upper gastrointestinal system endoscopy was performed while 123 patients consist of 51 male and 72 female was determined as IP. The prevalence of IP in patiens who underwent upper gastrointestinal endoscopy was 3.14% in our study. The majority of symptoms of those who had IP were laringopharyngeal reflux symptoms. Heterotopic gastric mucosa was fixed in 114 cases while 28 chronic inflammation, 9 esophagitis, 5 intestinal metaplasia, 4 glicogenic acanthosis were obtained as additional findings in pathological examinations.

**CONCLUSION:**

Heterotopic gastric mucosa in the proximal esophagus is a frequent finding if the endoscopist is aware of this entity. The importance of IP is the increasing number of cases of neoplastic transformation. Symptomatic patients should be treated and should be considered of the complications of heterotopic gastric mucosa.

## INTRODUCTION

Since the first described by Schumidt in 1805 as cervical inlet patch for oesophagus, heterotopic gastric mucosa have been reported in duodenum, jejunum, cystic duct, ampulla of vater, gallbladder and anus (1-6). Inlet Patch (IP) or rarely referred as “cervical inlet patch” (CIP) is characterized by an island of heterotopic columnar gastric mucosa that is placed in proximal oesophagus and commonly located just below the upper oesophagus sphincter (7).

Although ethology and pathology of IP could not be proved significantly, the incidence of this lesion has been reported with a high proportion of 4–10% (8-12). Also IP can be seen in pediatric population and clinical manifestation usually different from the adults. Heterotopic gastric mucosa is widely thought to be a congenital in nature. Recent studies reported that IP might be an acquired condition (7, 13). Because of the capable of mucin and/or acid production of IP, laringopharyngeal reflux symptoms with heartburn or/and dysphagia might be the clinical consequence. Most of the symptoms are not intensive and usually the management depends on the type and severity of symptoms. The clinical significance of IP is confused due to the limited published studies in the literature.

In upper gastrointestinal system, the histological changes such as atrophy, metaplasia, dysplasia and carcinoma; diagnosed and surveillance have become importance. In this study we aim to determine the frequency of IP and its association to clinical symptoms and draw attention to be aware of this heterotopic lesion.

## MATERIAL AND METHOD

This study was a prospective case series that gathered in two different institutions within 40 months. All patients with laringopharyngeal reflux symptoms underwent endoscopy by two experienced endoscopists between March 2009 and July 2012. The data was prospectively evaluated. In all cases, we analysed endoscopic findings of upper gastrointestinal system up to second part of duodenum including esophagitis, gastritis, bulbitis and lower esophageal sphincter deficiency. We compared the prevalence with those of patients that did not determine IP in the study period.

Patients were referred for endoscopy for a variety of reasons, principally for the evaluation of dysphagia and dyspepsia. In these patients, symptoms of globus sensation (lump in the throat), hoarseness, sore throat, frequent clearing of the throat, cough, heartburn, dysphagia and odynophagia were questioned at least 3 month duration prior to endoscopy.

### Esophagogastroduodenoscopy

All patients were signed written informed consent before endoscopic procedure. After an overnight fast, a routine esophagogastroduodenoscopy (EGD) was performed with a white light video endoscopy using high definition system (Fujinon endoscopic system 4400-Japan). All attempts were performed with the patient in left lateral decubitus position. For all cases topical anesthesia (xylocaine spray) and conscious sedation were performed. Conscious sedation was performed with midazolam (2­5 mg).

During all procedures the oesophagus was carefully surveyed and special attention was paid to the area of the upper oesophageal sphincter. This region was best examined when slowly withdrawing the endoscope, with repeated short inflations while rotating the instrument. Heterotopic gastric mucosa was defined as patches covered with salmon­red mucosa distinguishable from surrounding greyish­pearly coloured esophageal mucosa by well­defined margins. The size of the patches was determined by two different methods. Biopsy forceps was signed 1 cm intermittently as a ruler. Height and width (left to right and up to down) was measured. If there is a difference in diameter, bigger one was accepted. Additionally, if the difference was higher than 10%, the average diameter was taken. On the second methods endoscope device diameter is 10 mm and we can measure the lesion and compare the dimensions. We used these two methods to measure the lesions. In all subjects, the distance between the patch and the frontal incisor was recorded and the patch size measured under the guidance of the open biopsy forceps.

### Biopsies

Minimum of two biopsies were obtained from the IP and antral or/and gastric mucosa. All biopsies were taken from the lesion area and we did not damage the disease free area. The samples were taken using large cup and side opening forceps. Pathology performed to determine the presence of Helicobacter Pylori (H.P.) in all patients. All the specimens were evaluated by the same pathology service. The Sydney classification method was preferred for the histo-pathological evaluation of the biopsy samples (14). Diagnosis of Barrett’s oesophagus was based on histologically proven intestinal metaplasia in distal oesophagus. The presence of H. Pylori colony was identified using hematoxylin­eosin, cresyl violet, giemsa and silver stain. Biopsies of heterotopic gastric mucosa were classified into four main groups; oxyntic type, cardiac type, antral type, mixed type. The Histological Division of the Sydney System was intended to be a practical guideline highlighting which of the morphological features of gastritis in endoscopic biopsies should be documented, and how these might be graded. The final pathology report would then convey the type, severity and extent of the gastric pathology linked to the etiology where possible (14, 16, 24).

## RESULTS

In the study period 3907 upper gastrointestinal system endoscopies were performed while 123 patients consist of 51 male and 72 female was determined as IP. Mean age was 38.8 ± 12.5 (range 17-77). The prevalence of IP who underwent upper gastrointestinal endoscopy was 3.14% in our study. The majority of symptoms who have IP were LPR symptoms. Demographic characteristics and symptoms of patients who IP were detected are shown in Table 1.

**Table 1:** Demographic characteristics, prevalence of IP, and symptoms

Age (years)	38.8 ± 12.5 (min 17-max 77)
Gender (female/male)	72/51
Prevalence (%)	3.14%
Symptoms (%)	
Regurgitation	81 (65.8%)
Dyspepsia	68 (55.3%)
Dysphagia	48 (39%)
Globus	21 (17%)
Hoarseness	16 (13%)
Chronic cough	11 (8.9%)
Anemia	9 (7.3%)
Others	7 (5.7%)
Total	261^a^

a This is the total result of the symptoms. Because some of patients have multiple symptoms.

### Characteristic of lesions

**Figure 1:**
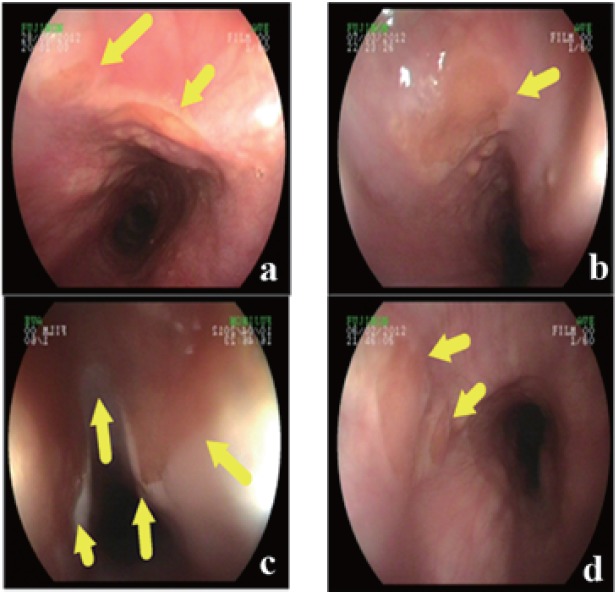
a, Two different inlet patch zone 14 cm distal from the incisors; b, Single inlet patch zone 15 cm distal from the incisors; c, A huge inlet patch zone 18 cm distal from the incisors; d, Two different inlet patch zone 13 cm from the incisor.

IP was identified as a discrete, pink or yellowish type lesion in the proximal esophagus, mean distance of the lesion from the incisors was 16.6 ± 3.1 cm (Figure 1). Mean diameter of the IP was 1.13 ± 0.5 cm. A secondary IP lesion was obtained in 11 patients that have 0.7 ± 0.3 cm mean diameter. The other lesions in addition to IP that detected in the course of endoscopy were gastritis in 98 patients, esophagitis in 43 cases, bulbitis in 17 cases and lower esophageal sphincter deficiency in 11 cases. Gastric and duodenal ulcer was diagnosed in 32 patients. 5 patients had normal endoscopic findings except IP.

### Histological findings

**Figure 2:**
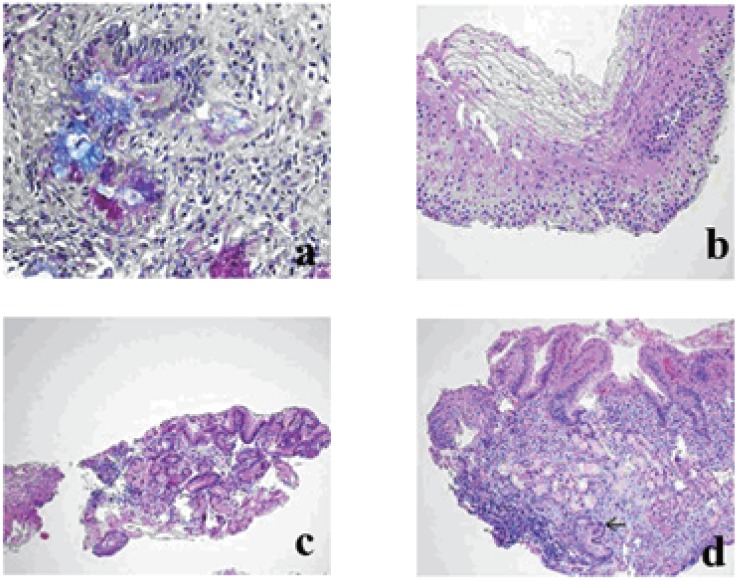
a, Intestinal metaplasia in the heterotopik gastric mucosa (PAS-AB pH 2,5 stain ×200); b, Glycogenic acanthosis (H&E ×200); c, Antral type heterotopic gastric mucosa. (H&E ×100); d, Heterotopic gastric mucosa composed of mixed oxyntic and antral type glands. Inflammation and intestinal metaplasia (on the bottom) (arrow) in the heterotopic mucosa are seen. (H&E ×100).

**Figure 3:**
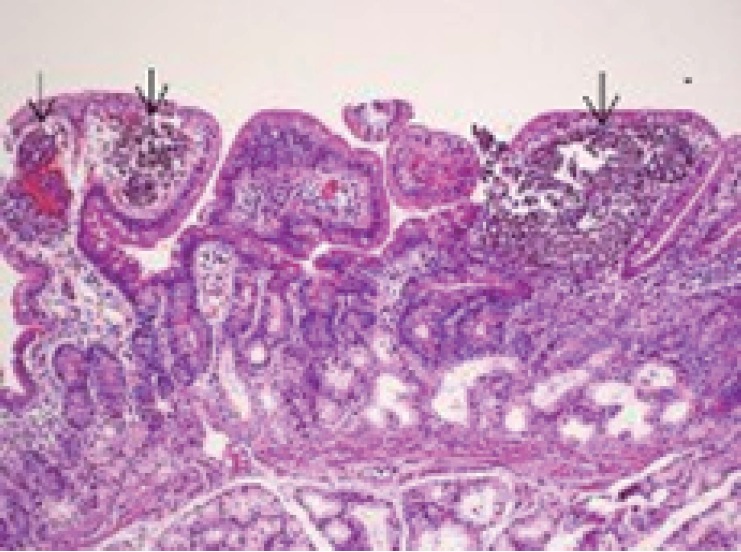
Metastatic malignant melanoma. Intralymphatic malignant melanoma cell clusters in the duodenal villi.(H&E ×100).

Biopsies from the IP and antrum and/or corpus were taken all of the IP patients. Heterotopic gastric mucosa was fixed in 114 cases while 28 chronic inflammation, 9 esophagitis, 5 intestinal metaplasia (Figure 2a), 4 glicogenic acanthosis (Figure 2b) were obtained as additional findings. Biopsy was unsuccessful in the remaining 9 cases because of the technical difficulties and patient status. Barrett’s esophagitis was seen only in two patients. These two patients were follow-up by clinic and endoscopic evaluations, frequently. There was no any atrophy, dysplasia or adenocarcinoma in the IP specimens. Cell types of IP were oxyntic type in 49 cases, cardiac type 34, antral type in 17 patients (Figure 2c), mixed type (oxyntic and antral) in 14 patients (Figure 2d). H.pylori colonization was found in 2 of 114 heterotopic gastric tissues. 58 of 123 patients had H.pylori in their antrum and/or corpus biopsies. These infected patients were treated by amoxicillin+clarithrpmycin+lansoprazole-combined therapy for 14 days. Intra lymphatic malignant melanoma cell clusters were determined in the duodenal villi from one of the pathologies (Figure 3). Histological properties of patients who IP were detected are shown in Table 2.

**Table 2:** Histological findings, The presence of H. Pylori colony was identified using hematoxylineosin, cresyl violet, giemsa and silver stain. Biopsies of heterotopic gastric mucosa were classified into four main groups; oxyntic type, cardiac type, antral type, mixed type

Heterotopic Gastric Mucosa	114
Chronic Inflammation	88
Esophagitis	41
Intestinal Metapylasia	5
Glycogenic Acanthosis	4
Barrett’s Esophagus	2
Total	138^a^
Cell Types	
Oxyntic type	49
Cardiac type	34
Antral type	17
Mixed type	14
Total	114
H. pylori	
Heterotopic gastric mucosa	2
Antrum or/and corpus	58
Total	60

a These is the total number because some patients have multiple symptoms.

## DISCUSSION

The presence of heterotopic gastric mucosa as an aberrant gastric epithelium in proximal oesophagus was first described in 1805 by Schmidt from autopsy examination (6). Other located areas for heterotopic gastric mucosa have been reported in duodenum, jejunum, cystic duct, gallbladder, rectum and anus (1-7). IP occurs most frequently in the postcricoid portion of the oesophagus at or just below the upper oesophageal sphincter (7, 15). Ectopic mucosa can also be found in the other parts including the distal part of the oesophagus (16, 17). There are three theories including congenital origin, metaplastic transformation and rupture of cystic glands that were proposed for the development of cervical inlet patch (7, 13, 14). Inlet patch is widely considered as a congenital anomaly, in literature. Since the development of the oesophagus at 24 week of gestation, squamous lining replaces the columnar lining from the middle oesophagus extending in both direction and this accounts for the postcricoid location of inlet patch (18). Authors proposed the ability of endodermal cells of the primitive gut throughout the gastrointestinal tract to differentiate and undergo hyperplasia or physical movement of the gastric epithelia due to unknown pathways (19, 20). The other theory is an acquired theory that depends on the chronic acid injury as seen in Barret’s esophagus. This drawback is responsible of the transformation of squamous lining to columnar (13). The other less common theory involves rupture of proximal oesophagus retention cystic glands (14). In this study we want to demonstrate the incidence of IP and show the clinical and histological importance of the lesion.

Although the reported prevalence of IP generally varies from 0.1 to 10%, the incidence has reported up to 70% in autopsy studies (7, 16). In our case series, we found the incidence 3.41% that correlated with the literature. Even within our ethnic origin, IP has been reported in 1.6- 3.6% of cases in two studies (21, 22). The relatively high prevalence of inlet patch in some studies compared to others may be explained by the special interest of certain endoscopists who intentionally look for it (23). Even though IP is mostly asymptomatic and is detected incidentally during the evaluation for other gastrointestinal complaints, rarely pain and dysphagia may be described by patients (24, 25). Globus sensation, hoarseness, odynophagia, dysphagia, or oropharyngeal burning (regurgitation) may be the symptoms that occur in 6.2 to 20% of cases (26). These symptoms are usually related to the acid release that produced by the patch. Neuman et al reported the largest chain that involved 487,229 cases; they showed that dysphagia or odynphagia, regurgitation and globus were significantly more common in patients with cervical inlet patch (27). It is reported that most of the symptoms were mild. In our case series; the most common symptom was regurgitation, which was seen in 81 (65.8%) of 123 patients. It must be considered that in problematic cases complications related to acid secretion such as esophagitis, ulcer, web, stricture and fistula may produce symptoms such as chest and throat pain, globus sensation, chronic cough and shortness of breath (18, 28).

It is difficult the detected the heterotopic gastric mucosa during routine endoscopy. The endoscopist should be aware of this lesion while around of the upper esophageal sphincter. At endoscopy, the lesion appears salmon-colored and round or oval with a flat, slightly raised, or depressed surface and may have heaped margins most often on the lateral or posterior walls typically a few centimeters distal to the upper esophageal sphincter (1-12). The lesion will be seen more often during the endoscopy while withdraw the scope very slowly through the upper sphincter. Contractions of the upper oesophageal sphincter during endoscopy make inspection and biopsy of this area difficult (29). It is seen that the distance of the location of IP from the incisors vary from 16 to 21 cm (7, 12, 24). In our series the located area of IP is an average of 16.6cm. The ultimate diagnosis of inlet patch is confirmed via endoscopy with biopsy. In our study due to the technical problems, histologic confirmation could not prove in 9 patients. In the pathologic evaluation of the biopsies the most common histological type is the oxyntic or cardiac type mucosa followed by the antral mucosa (7-10). In our series the most common type is oxyntic type (43%) that parallel to literature.

The size of inlet patches can vary from microscopic to 5 cm. IP can be round with a flat, slightly raised, or depressed surface and may have heaped margins most often on the lateral or posterior surfaces (26). In our study mean diameter of IP was measured as 1.13 ± 0.5 cm and located especially lateral and posterior surface of the oesophagus that accordance to literature. Takeji et al reported that ectopic gastric mucosa in the oesophagus to be more common in men than in women (30). Our gender results were against to this result. Although single patch is detected commonly in literature, multiple patches can be found within close proximity of other patches (21, 31, 32). We observed eleven patients that have more than single lesion. These findings were in accordance with the literature.

The clinic significance of IP is mainly acid related complications and neoplastic transformations. Inflammatory and pathologic changes such as atrophy, intestinal metaplasia, dysplasia and carcinoma even angiodysplasia have also been reported (16, 21, 33). Stricture, erosion, ulceration, cystic dilation of the glands, fibrosis, intestinal metaplasia, web, perforation and polyps were reported in the literature as complications of heterotopic gastric mucosa (33-40). Intestinal metaplasia has been reported in association with adenocarcinoma developing inlet patch (33). Alagozlu et al encountered only one adenocarcinoma in 64 patients with heterotopic gastric mucosa and estimate the incidence of malignancy ranges 0 to 1.56% (34). We determined intestinal metapylasia additional to IP only in five of cases but no any adenocarcinoma and dysplasia was seen. Relationship with glycogenic acanthosis and IP has not been defined yet. Glycogenic acanthosis are small discrete elevations in the esophageal mucosa. It has been known that glycogenic acanthosis is a common condition, its frequency is 3.5%, and may be associated with reflux esophagitis (41). It is endoscopically recognized as slightly elevated iodine-positive small areas and these lesions are confirmed by PAS staining. In consequence of symptoms were improved by anti-reflux therapy, only four cases were detected in our series.

Helicobacter Pylori (HP) is a well-known spiral or curved gram-negative non–spore-forming bacillus responsible for chronic inflammation. Ectopic gastric mucosa of the inlet patch is an ideal location for HP colonization (42). HP positivity of heterotopic gastric mucosa in oesophagus has been reported 0 to 86% when HP is present in the stomach (11, 21, 26, 31, 42). Although the role of HP in IP remains uncertain, it is estimated that it can cause histological changes similar to seen in stomach. In our study among 114 patients with inlet patch, 2 were positive for H pylori while 58 of corpus and/or antrum biopsy positive with HP. Eradication was obtained in these infected patients after that 14-days-combined therapy.

The relationship between heterotopic gastric mucosa and Barrett’s oesophagus has been controversial. There are also reports that IP is associated with an increased risk for Barrett’s oesophagus (13, 43). Traditionally, Barrett’s oesophagus is considered a distinct entity from oesophageal inlet patch. In contrast to this positive correlation, it is asserted that have not been found any correlation with Barrett’s oesophagus and IP (16, 17, 22). Barrett’s oesophagus is an acquired precancerous lesion and the cell origin probably involves multipotential undifferentiated cells. Up to half of all patients with cervical inlet patch have concurrent Barrett’s oesophagus in some reports (13). In this current study, we have observed 2 patients 1.75% coexistence between Barrett’s esouphagus and IP. (Only 2 patients with IP have Barrett oesophagus).

Unfortunately; in consequence of low incidence and lack of information on its prognosis, consensus guideline for surveillance of IP has not been established, currently. Incidental identification of IP does not require additional specific treatment unless significant respiratory symptoms are present. These symptoms should be sought by direct questioning if not reported. These lesions must be evaluated by histological examinations for detection of unsuspected findings or malignancy. There is no suggestion to treat the asymptomatic inlet patches. Affected individuals who are symptomatic may find relief with the use of proton pump inhibitors. Generally it is considered that complication of the IP should be treated such as the strictures and webs treatment with serial dilatation (7, 21). Endoscopic mucosectomy, ablation of inlet patches and surgical resection has been shown for treatment and radical surgical attempt has been used to successfully treat inlet patch dysplasia or malignancy (7, 21, 26).

## CONCLUSIONS

We conclude that the presence of heterotopic gastric mucosa in the proximal oesophagus is a frequent finding if the endoscopist be aware of this entity. Although the manifestations are commonly asymptomatic, symptomatic cases will be mingling with the other upper digestive disorders. The importance of IP is the increasing number of cases of neoplastic transformation. Symptomatic patients should be treated and should be considered of the complications of heterotopic gastric mucosa. Currently, there are still many point of IP such as manifestation, natural history and properties of mucosal changes that are not well understood.
